# Study Protocol for a Qualitative Research Project Exploring an Occupational Health Surveillance Model for Workers Exposed to Hand-Intensive Work

**DOI:** 10.3390/ijerph17176400

**Published:** 2020-09-02

**Authors:** Kristina Eliasson, Peter Palm, Catarina Nordander, Gunilla Dahlgren, Charlotte Lewis, Therese Hellman, Magnus Svartengren, Teresia Nyman

**Affiliations:** 1 Department of Medical Sciences, Occupational and Environmental Medicine, Uppsala University, Uppsala University Hospital, 751 85 Uppsala, Sweden; peter.palm@medsci.uu.se (P.P.); Therese.hellman@medsci.uu.se (T.H.); magnus.svartengren@medsci.uu.se (M.S.); teresia.nyman@medsci.uu.se (T.N.); 2 Occupational and Environmental Medicine, Lund University, 227 33 Lund, Sweden; catarina.nordander@med.lu.se; 3 Department of Public Health and Clinical Medicine, Section of Sustainable Health, Umeå University, 901 87 Umeå, Sweden; gunilla.dahlgren@umu.se (G.D.); charlotte.lewis@umu.se (C.L.)

**Keywords:** occupational health service, exposure assessment, musculoskeletal disorders, ergonomists, legislation

## Abstract

The objective of this study protocol is to describe the development of a process model for occupational health surveillance for workers exposed to hand-intensive work (the HIW-model), and to describe the studies that will explore the model. The studies are designed to: (1) explore stakeholders’ experiences of the model, and (2) explore if, and how, the model affects actions for reduction of exposure to hand-intensive work. The study protocol presents a research project that is described as two studies. The first study will explore company representatives’ and ergonomists’ experiences of the execution of the HIW-model and its various components concerning feasibility and values. Semi-structured interviews will constitute the data source. The second study will explore whether the execution of the HIW-model leads to work environmental changes, such as actions for reduction of exposure to hand-intensive work, and whether these potential actions are based on the ergonomist’s feedback of the exposure assessment and the medical health checks. A mixed method approach will be applied, in which the data sources will be comprised of semi-structured interviews, questionnaires, and documents. The project is expected to generate knowledge regarding the values of the HIW-model. The project is anticipated to shed light on factors that facilitate or impede execution of the model from the different stakeholders’ perspectives; the employer’s as having the legal responsibility for the work environment, and the occupational health service consultants’, being the work environment experts supporting the employers.

## 1. Introduction

This paper presents a study protocol that aims to describe the development of a process model for occupational health surveillance for workers exposed to hand-intensive work, and to describe the research project and the studies that will explore the model. Occupational health surveillance is a legislative approach for preventing work-related ill-health. In Europe, the European Framework Directive (1989/391/EEC) establishes the principles for managing health and safety in workplaces. The European Directive is a framework directive and constitutes the minimum requirements of the legislation. The EU member states can adapt the directive to their respective national regulations for incorporation in the country’s work environmental legislation. Each individual employer has the responsibility to ensure the health and safety of their employees, and to make sure that the workplace meets the requirements stipulated in the national work environment legislation [1,2]. However, employers can contract external work environment expertise, such as the occupational health service (OHS), for support regarding work environmental issues, such as risk assessment, occupational health surveillances, and rehabilitation.

A keystone in occupational health and safety management is periodical risk assessments of exposures in the workplace. Exposures that are identified as hazardous shall be managed by taking actions to reduce the exposure levels in order to minimize the hazard and to prevent work-related ill-health [1,3], something that in Sweden is described in the Swedish Work Environment Authority’s (SWEA) provision on systematic work environment management [3]. A supplementary approach for preventing work-related ill-health is periodical occupational health surveillance. Occupational health surveillance encompasses medical health checks tailored to specific hazardous exposures [4,5]. The combination of the two approaches, periodical exposure assessments and periodical medical health checks, provides a comprehensive presentation of the relation between work-related exposures and ill-health among workers [4].

In Sweden, as in most European countries, medical health checks have traditionally targeted chemical or physical workplace exposures. However, although musculoskeletal disorders (MSDs) are a major cause of sick leave and work disability in Europe [6,7], there are very few occupational health surveillance checks implemented which target exposures that are considered to be associated with an increased risk of developing MSDs.

In 2013, the SWEA began to revise the provision on occupational health surveillance, AFS 2005:6 [8]. One change was that the SWEA considered introducing a new medical health check, targeting workers exposed to hand-intensive work. The incentives behind this were both the increasing trend of carpal tunnel syndrome in Europe [6], and that previous research exploring the potential associations between hand-intensive work and risk for MSDs in the neck and upper extremities had concluded that workers in occupations with high levels of exposure have a higher risk of developing MSDs [9,10,11,12,13,14,15,16]. Exposure to hand-intensive work, which comprises rapid wrist movements towards the end range of motion, in combination with forceful exertions, are common in many different sectors. There is for example a high incidence of MSDs related to hand-intensive work in sectors such as slaughtering, assembly, packaging, grocery, hairdressing, and cleaning [15,16,17,18]. 

Even though there are legislations and regulations regarding risk management and occupational health surveillance implemented all over the world, research points to a lack of knowledge and understanding among managers when it comes to occupational health and safety management principles [19]. Previous studies from a Swedish context regarding medical health checks (regardless of type of exposure) have shown that employers rarely consult their contracted OHS-provider either for exposure assessments or for medical health checks. The results from these studies show that the OHS-providers interpreted this as a lack of knowledge among employers regarding their legal responsibilities, as expressed in the SWEA provisions [20,21]. 

In another Swedish study, the OHS-providers reported that even when the periodical medical health checks were performed as stipulated in the provisions, the connection with exposure assessments were lacking [22]. Consequently, the results from the medical health checks seemed rarely to result in any work environment actions aimed at reducing the exposure, neither on a group level nor on an organizational level. This indicates a knowledge gap among both the employers and the OHS-providers regarding how to interconnect the exposure assessment and the medical health check. Furthermore, it indicates that occupational health surveillance is seen as a separate entity and not as an integrated part of the occupational health and safety management system. The medical health checks are often regarded as activities not linked to the exposure in the work environment. It seems the legislative provisions do not provide enough incentives to integrate information of both the work environmental exposures as well as information of workers health into the risk management process. Hence, there is a need of guidance, for OHS-providers as well as employers, about the interconnection between work exposures and medical health checks, and how they should be integrated in the occupational health and safety management system [21,22]. 

With awareness of this knowledge gap, and in consideration of the new medical health check targeting hand-intensive work, the SWEA turned to the authors, TN, PP, CN, and MS, with a request to develop a process model for occupational health surveillance of workers exposed to hand-intensive work (HIW-model). The aim was to construct a model that could be used by employers (as having the legal responsibility for the work environment) as a source of support, but that was also useful for the OHS (being independent experts supporting employers regarding the work environment and health-related issues). The occupational health service is a multi-professional organization. The relation between the company and the OHS-provider can be either that of an “in-house” department within the company, or that of an external resource acting as a consultant. Within the OHS, the expertise relating to hand-intensive work and musculoskeletal disorders foremost lies with the ergonomist, who in Sweden often has a background as a registered physiotherapist, with additional education within ergonomics.

The objective of this study protocol is to describe the development of a process model for occupational health surveillance for workers exposed to hand-intensive work, and to describe the research project and the studies that will explore the model.

### 1.1. Theoretical Framework for the Development of the Process Model 

Andersen et al. (2019) concluded in a review that legislative and regulatory policies may reduce injuries and fatalities, and improve compliance with occupational health and safety regulations. Furthermore, they also identified a research gap concerning regulations targeting MSDs [23]. Gaps between research, decision-making, and practices are well known problems. In order to achieve practical use concerning, e.g., new regulations, these need to be transferred into practice by active knowledge translation. Knowledge translation describes the process of putting knowledge into action, closing the gaps between research and practice [24]. The knowledge-to-action (KTA) framework, developed by Graham and colleagues, guides the application of knowledge [25]. The framework provide a map to guide the process of how to move the not-yet-applied knowledge to applied practice [25,26]. The knowledge-to-action translation is an iterative, dynamic, and complex process, which should take into consideration the various stakeholders involved [24]. The KTA framework has two components: the knowledge creation and the action cycle. The knowledge creation guides the process for production of knowledge. In the knowledge creation process knowledge is being further distilled and refined throughout the process so that it results in a product/tool more useful for the end user/stakeholders. The knowledge creation process for the development of the process model for occupational health surveillance for workers exposed to hand-intensive work is presented in this study protocol. 

### 1.2. The Knowledge Creation Process for an Occupational Health Surveillance Model for Hand Intensive Work

As indicated in previous studies both employers and OHS (in this case ergonomists) need to be addressed in the development process [20,21,22]. In order to construct a model for occupational health surveillance that facilitates the collaboration between the employer and the OHS-ergonomist, and which leads to integration of occupational health surveillance in the occupational health and safety management system, the process and the components need to be tailored and recognizable for the employer, as well as for the OHS-ergonomist. 

**Figure 1:**
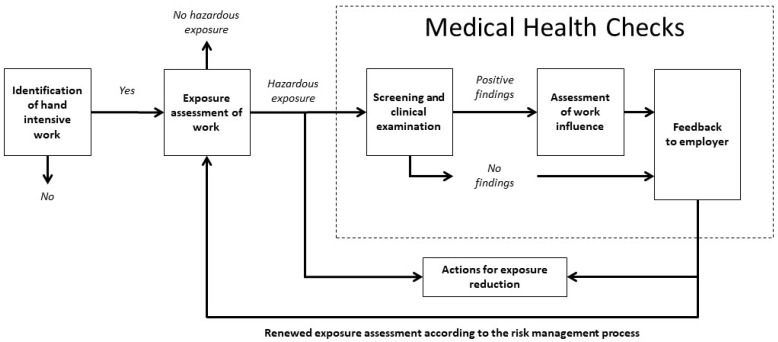
The model for occupational health surveillance for workers exposed to hand-intensive work (HIW-model).

Therefore, a feasible starting point for the development of the process model was to take inspiration from core components in a risk management process: identification, assessment, control, and monitoring of workplace hazards [3]. The authors, TN, PP, CN, and MS constructed a draft of the model and discussed the draft with physicians and ergonomists at SWEA. The model was judged against the Swedish national work environment legislation and the applicable provisions [3,8,27]. After revisions, the model was presented to ten experienced ergonomists who reviewed the model. The ergonomists gave their comments and feedback on the model in telephone interviews conducted by TN. The proposed model was then revised before the final step in the knowledge creation phase, where the model was presented and discussed at a workshop with stakeholders from relevant trade organizations and trade unions, such as the trade and commerce sector, metal industry, the engineering industry etc. The final revised model is presented in Figure 1. 

### 1.3. Description of the Components of the Model 

*Identification of hand-intensive work*: The initial component in the model aims to crudely identify whether the workers at the company in question are exposed to hand-intensive work or not; thus, no extensive expertise in ergonomics is needed. It is intended to be performed by company representatives, for example, a manager together with a safety representative, and should render an answer to the question: Are the workers exposed to hand-intensive work for more than four hours/day? Yes or No. Hand-intensive work will be defined as: continual rapid wrist movements towards the end range of motion, in combination with forceful exertions [27]. The four hours/day or more duration limit of exposure to hand-intensive work can be considered to correspond to a major part of the work day, and is also based on the threshold limit value described by the American Conference of Governmental Industrial Hygienists regarding threshold limit value for hand activity [28]. 

If the identification reveals that there are workers exposed to hand-intensive work for more than four hours/day, or if there is an uncertainty regarding the exposure, the analysis progresses on to the next component in the model.

*The exposure assessment of work*: For execution of this component of the model, more in-depth knowledge in ergonomics is required as an exhaustive exposure assessment of hand-intensive work needs be done. The company may need to turn to their contracted OHS-provider for the execution of the exposure assessment. The assessment should take into account the duration, the frequency, and the intensity of the exposure. Based on the characteristics of the work/work-tasks, suitable methods should be used for exposure assessment, which may encompass observational assessment methods and/or direct technical recordings in different combinations [29,30]. Ultimately, the assessment should give an estimation of the dose of the exposure and whether this dose implies a risk for ill-health. If the results of the assessment disclose that a reduction of exposure is needed, actions should be initiated as part of the periodical risk management process [3]. However, if the exposures cannot, at an early date, be reduced to an acceptable level, as a next course of action exposed workers should be offered medical health checks.

*The medical health check* is intended to reveal additional information as to whether the hazardous exposure is associated with any musculoskeletal symptoms, and encompasses the following three parts: (1) Screening for musculoskeletal symptoms and prior ill-health in the exposed worker. Workers with positive findings are offered a clinical examination of hands, arms, shoulders, and neck. (2) An assessment of if, and how, the exposures (assessed in the earlier step) are associated with positive findings of musculoskeletal symptoms in exposed workers, and (3) feedback to the employer, with the intention to start a dialogue with the employer concerning actions on both individual, group, and/or organizational level.

The person who performs the three parts of the medical health check should, according to the model, have:

- good knowledge of systematic work environment management;
- good knowledge of the worker’s exposure and working conditions;
- clinical competence for examination of the musculoskeletal system; and
- competence to assess whether the hand-intensive work can cause problems in the neck, shoulder, arm, or hand.

*Actions for exposure reduction:* The results of the exposure assessment, together with the results of the medical health check, constitute a comprehensive picture of if, and how, the previously assessed exposure is related to the potential musculoskeletal symptoms in the workers. Based on the results from both the exposure assessment and the medical health checks, exposure-reducing actions should be initiated. 

### 1.4. Description of Stakeholders Involved in the Execution of the Model

The model is intended to support a participative approach. The execution of the model is a collaborative process between company representatives (e.g., first line managers, health, safety, and environment managers, who represent the employer, and safety representatives, who represent the workers) that are affected by the new occupational health surveillance regulation targeting hand-intensive work, and the ergonomics expert from the OHS-provider. Since OHS-ergonomists in Sweden usually have a background competence as registered physiotherapists, with further education in ergonomics, they thus hold the competence to perform both the exposure assessment and the clinical examination in the medical health check. 

## 2. Materials and Methods for the Exploration of the Application of the Process Model

The model is planned to be explored in two studies. The objective of study 1 is to explore company representatives’ and OHS-ergonomists’ experiences of the execution of the HIW-model as a whole, and its various components concerning values and feasibility. Seen from the perspective of the KTA-framework, this study is related to the action cycle [25], and will focus on the application of the model, such as the adaption of the model to local context, and exploration of facilitating or hampering determinants. 

The objective of study 2 is to describe and explore if, and how, the HIW-model affects actions for reduction of exposure to hand-intensive work in the work place, as well as any perceived changes in workload and pain in the neck, and upper extremities, among the workers. In relation to the KTA-framework [25], study 2 will explore the outcomes of the use of the HIW-model. 

The Regional Ethical Review Board in Uppsala has approved the research project (project reference number 2017/274). 

### 2.1. Description of the Target Population and the Recruitment Procedure for the Project

To conduct the project, companies will be selected through purposive sampling [31] of companies with hand-intensive work tasks. The Occupational and Environmental Medicine Clinics at Uppsala, Umeå, Lund, and Gothenburg will assist with tips regarding companies that can be relevant to contact. The intention is to include a heterogeneous group of companies, representing different sectors, varying in size, and located in different geographical regions (in Sweden). Many workers exposed to hand-intensive work are also operating hand-held vibrating tools, and are hence exposed to hand-arm vibration. Exposure to hand-intensive work often coexists with contact with hand-held vibrating tools and exposure to hand-arm vibration. Occupational health surveillance with medical health checks targeting exposure to hand-arm vibration has been regulated in Sweden since 2005, in the provision on occupational health surveillance, AFS 2005:6 [8]. Therefore, in order to explore the process model, on employers that until now have had little or no experience of medical health checks, the project will mainly target populations that are exposed solely to hand-intensive work according to the definition above. Sectors that are relevant to target are, for example, different types of assembly, painting, cleaning, food handling (catering), dental technology, and companies with hand-intensive manual material handling. The company should have at least 8–15 workers exposed to hand-intensive work that are willing to take part in the occupational health surveillance. The project will be introduced to company managers in the selected sectors. They will be contacted via telephone or e-mail and provided with both oral and written information about the research project. 

A prerequisite for participation is that the company has an ongoing agreement with an OHS-company, and that an ergonomist from the OHS-provider can be assigned to the project. Furthermore, the services linked to the project and provided by the ergonomist should be charged according to the existing financial agreements between the company and the OHS-provider, where the employer is charged by the OHS-provider for services rendered. When these criteria are met, the researchers will contact each companies’ OHS-ergonomist and provide oral and written information. 

The recruitment will be completed when between 10 and 12 companies, representing a variation among sectors, have accepted participation. 

When selected companies have agreed to participate, every company will be asked to form a project group that consists of relevant company representatives (e.g., first line manager, health, safety, and environment manager, safety representative). The purpose of the companies’ project groups is to be responsible for exploring the HIW-model in their respective company. In order to explore different contextual courses of action, each company will, based on their preconditions and organization, decide by themselves which, and how, many representatives they want to involve in the project. However, they will be informed that at least one manager, with responsibility for the work environment, and one safety representative should be participating in the project group. The size of the project group may vary between two to five individuals. 

The project groups will also be supplemented with the ergonomist from each company´s contracted OHS-provider. The role of the ergonomist in the project will be to execute the exposure assessment, medical health checks, and report the results to the company representatives.

After each company has decided the composition of the project group, every group member will be given oral and written information about the project, and will be informed that participation is voluntary and that they are free to withdraw at any time. Informed consent forms will be distributed for signature.

The project group from each company shall select a department within their company, which shall be the subject of the occupational health surveillance for hand-intensive work. In order to be manageable, with regard to the time frame for the project, there will be a restriction in size of department, of approximately 8–15 workers. The workers will be informed about the project, and that they will participate by being offered to undergo a clinical examination, and also that some workers might be involved in the exposure assessment. Furthermore, they will answer three questionnaires during the research project. The participating workers will be given written information about voluntary participation, and consent forms will be distributed and signed together with the base-line questionnaire.

### 2.2. Description of the Study Process 

The study process starts with a start-up day in three different regions. An overview of the research project will be presented for the project groups, followed by presentation of the HIW-model (Figure 1). Thereafter, a short lecture will be given, including information about how to plan and execute the exposure assessment. Information about observational exposure assessment tools, as well as information concerning the scope and content of the clinical examination will also be provided. Furthermore, a proposal for a short feedback report template will be presented. The template summarizes the findings from the different components of the model, e.g., number of exposed workers, number of exposed workers with symptoms in the upper extremities and/or neck, work environment exposures related to symptoms, and proposals for actions to be taken. The ergonomists will be given information because, depending on differences in organizations, they might need to tailor the execution of the exposure assessment and medical health checks to suit “their” company. This means that, based on their expertise and experience, they need to choose exposure assessment tools that are suitable for their context, and whether the execution of the clinical examination can be done following a structured method or not. 

Following the lecture, there will be time scheduled for the project groups to plan and design their respective processes for the conduction of the occupational health surveillance. They will be given some specific issues to consider:

- Work-tasks for exposure assessment;
- Suitable exposure assessment tools;
- Clinical examination protocol/method;
- Timeline for execution of exposure assessment and medical health checks;
- Target group for feedback of the results and action proposals;
- Work process regarding action proposals;
- Responsibility for action proposals;
- Quality outcomes for evaluation of risk reducing actions

### 2.3. Description of the Expertise and Specific Training Given to the Ergonomists 

The ergonomists who will execute the exposure assessment and medical health checks will be invited to participate in a one-day course in the structured examination method, “health surveillance in adverse ergonomics conditions,” which includes a screening part and an in-depth clinical examination part, of the neck and upper extremities [32]. There will be no requirements on the ergonomists to use this examination method. Regarding the exposure assessment (and choices of exposure assessment tools), no specific education will be offered to the participants, except for the information shared in the lecture at the start-up meeting. 

## 3. Description of the Research Project

### 3.1. Study 1: Exploring the HIW-Model and Its Various Components in Terms of Feasibility and Values 

#### 3.1.1. Study Design

Study 1 will use a qualitative research methodology with an exploratory design. Since the objective of the study is to explore the end-users’ experiences of the use of the model, a qualitative research design is suitable [31]. Semi-structured interviews will constitute the data sources. 

#### 3.1.2. Aim 

The aim of study 1 is to explore company representatives’ and OHS-ergonomists’ experiences of the execution of the HIW-model and its various components, in terms of feasibility and values, and to identify factors that facilitate or impede the execution of the model. 

#### 3.1.3. Study Participants and Data Collection 

As described in the section *“Target population and recruitment procedure of the project”* approximately 10–12 companies will participate in the study. In order to get a broad picture of how the HIW-model is experienced and what factors promote implementation, the perspectives from the companies as well as from the ergonomists need to be collected.

Information from both safety representatives and managers in different positions are valuable. Therefore, the company representatives from each company’s project group will be interviewed in order to collect experiences from their point of view. In order to explore the experiences of the executor of the HIW-model, each ergonomist will be interviewed. 

Data will be collected through individual interviews and focus group interviews on two occasions during a six-month period, the distance between interview one and interview two depends on the individual process in each company. However, to ensure that the process will have been completed, the minimum time period between the two data collection points will be at least four months. 

Interview one will be held as an individual telephone interview with each company representative and each ergonomist. Approximately 40–48 interviews will be conducted (three company representatives from 10–12 companies and one ergonomist/company). The interviews will be conducted shortly after the execution of the exposure assessment and the medical health checks. Interview one will explore the different components of the model (from screening to medical health checks). The interview will start with one broad question concerning each of the components in the model (Figure 1), followed by probing questions. For example one initial question will be: “Please, tell about the exposure assessments?” Probing questions will then explore in more detail how this was executed, who was involved, what tools were used etc. Next, broad questions will be about the medical health checks, how those were executed, what worked well, and what needs to be improved. 

Interview two will be conducted as a face-to-face focus group interview, with each company’s project group. Individual interviews will be held with the ergonomists. The second interview will be held by an interviewer-pair, with one interview moderator and one observer that asks supplementary questions. The second interview will explore the informants’ experiences of the model as a whole; for example, questions regarding feasibility, usability, and value. Furthermore, the interview will explore facilitators and barriers in the work process. The questions will be, for example: “Please, tell us what you think about the HIW-model”. “Did the occupational health surveillance for hand-intensive work bring any value? “What aspects were important from your point of view for a successful process?”

All interviews will be semi-structured and follow an interview guide. The interview guide will be modified somewhat between the informants, depending on if it is the company representatives or the ergonomist that is interviewed. However, the guide will have the same base and content. The interview guide will be tested before the first interviews. 

Documents will also be collected, for example, the planning document that the company representatives generate together with their ergonomist at the start-up meeting. The ergonomists will be asked to keep a logbook, which shall include what they are doing in the process, how many hours each phase takes, which tools are used for the exposure assessment, and how they conduct the medical health checks. 

#### 3.1.4. Analysis

The interviews will be transcribed verbatim, and the manuscripts will be analyzed using qualitative content analysis [33,34]. In total, there will be approximately 60 interviews for analysis. In order to strengthen the trustworthiness of the analysis and minimize the risk that the analysis is conducted from one person’s understanding, several of the researchers will be involved in the analysis [35,36]. The interviews representing the companies’ perspectives will be analyzed by one pair of researchers, and the interviews representing the ergonomists’ perspective will be analyzed of by another pair of researchers. The reason for separating the analysis is to avoid the researcher being affected by the experiences of the other party (company or ergonomist). 

The analysis encompasses several steps. First, the material will be read through to create an overall understanding of the data. Thereafter, the coding process will start; meanings in the text will be coded related to the study aim. The coded meaning units will be close to the participants’ own wording, and labelled into different sub-categories and categories. The coding process is inspired by Graneheim and Lundman, and Elo and Kyngäs [33,34,35]. The software program NVivo 12 (QSR International Pty. Ltd., Doncaster, Australia) will be used to organize and store the data and facilitate a deeper analysis. When the initial categorization is made of the material, the categories and codes will be discussed in the research group in order to increase credibility [36]. The researchers who have not been involved in the coding process will bring forth new questions and other ways to interpret the material. After discussions, the material will be further analyzed, and codes and categories discussed in the research group in order to reach a consensus and to finally form the result. 

### 3.2. Study 2: Exploring if and How the HIW-Model Affects Actions for Reduction of Exposure to Hand-Intensive Work

#### 3.2.1. Study Design

This study will have a mixed method approach. Questionnaires, documents, and interviews will constitute the data sources. By using a mixed method approach data are triangulated which increases the trustworthiness in the findings [31,37]. 

#### 3.2.2. Aim 

The aim of study 2 is to explore whether the execution of the HIW-model led to any changes in the work environment, such as actions for reduction of exposure to hand-intensive work, and what kind of actions have been taken. Furthermore, to explore whether these potential actions were initiated based on the ergonomist’s feedback of the exposure assessment and the medical health checks. 

A second aim is to explore the workers’ perceptions of potential changes in the work environment, as well as any perceived changes in workload and pain in the neck and upper extremities.

#### 3.2.3. Participants and Data Collection 

The company representatives from the partaking companies and their respective ergonomist will be interviewed regarding if, and how, execution of the model led to any work environment actions at the work place. The interviews will be conducted via telephone approximately 18 months after the second interview in study 1 (“Exploring the HIW-model and its various components in terms of feasibility and values”). The choice of time to follow up is to allow enough time so that exposure reduction actions may have been implemented. The interviews will be semi-structured and follow an interview guide. 

The basis for the interview guide will be the written reports that the ergonomists provide to the companies after completion of the occupational health surveillance. According to the instructions given in the model, the report will contain the results of the exposure assessment and medical health checks but also any proposals for actions to reduce the hand-intensive exposure in the work place. Questions that will be posed in the interview include, for example, why actions have been taken or not, how actions are being implemented, and tentative effects of those actions. Other areas of interest are, which stakeholders have been involved in the actions, whether there has been any worker participation, or whether the ergonomist has partaken. 

To evaluate the workers’ perceptions, the study population will consist of the approximately eight to fifteen exposed workers from each of the participating companies. The project group in each company will select the group of workers (see section “Description of target population and recruitment procedure for the project”). In all, the estimated study population will include approximately 150 exposed workers. Questionnaires will be distributed to the workers on three occasions: at baseline, at 6-months, and at the 15-month follow-ups. Questions will cover the areas of work-related exposures, and perceived pain in the neck and upper extremities. The questions concerning pain will be formulated according to the validated “standardized Nordic questionnaires for the analysis of musculoskeletal symptoms” [38]. Questions regarding perceived workload as well as questions concerning the worker’s perception of the company’s occupational health and safety management system will be included, and phrased in accordance with questions in the bi-annual SWEA surveys regarding work environment [39]. In the follow-up questionnaires, open-ended questions about any actions following the execution of the model will be included. Members of the research group will administer the baseline questionnaire directly to the workers in conjunction with the information meeting. The second and third questionnaires will be distributed as paper questionnaires, and will be sent by mail directly to the individual workers. 

#### 3.2.4. Analysis

For the exploration of potential changes in work environment, open-ended questions in the workers’ questionnaires, and interviews with the company representatives and the ergonomists will be included in the analysis. The interviews will be transcribed verbatim. All texts will be analyzed using qualitative content analysis [33,34], the analysis process will be executed as described in the analysis section above, in “study 1: exploring the model components and process”. 

The expected sample size is approximately 150 individuals, and the expected response rate of the baseline questionnaire is 75%. With this relatively small sample size, and an expected decreasing response rate in the follow-up questionnaires, the statistical power will not be sufficient for parametric inferential statistical methods. Descriptive statistics will be used to report on the workers’ perceived workload and prevalence of perceived pain in the neck and upper extremities, and be based on the three repeated workers’ questionnaires (baseline, 6-months, and 15-month follow-ups). Non-parametric statistical tests will be used to investigate, e.g., potential differences in perceived workload and prevalence of perceived pain between baseline and follow-ups.

## 4. Discussion

This study protocol describes the development of a process model for occupational health surveillance for workers exposed to hand-intensive work. Furthermore, it describes the two studies aiming to explore the feasibility and values of the model from three different perspectives; the employers’, the providers’, and the workers’. The results from those studies are expected to provide answers regarding the usability of the model and how it should be further developed to form the design for implementation guidelines as well as for intervention studies. 

The focus in the model is the interconnection between the exposure assessments and the medical health checks. Collecting information from the different stakeholders’ perspectives regarding the content of the model and its various components, as well as the execution of the model as a whole, will provide information regarding implementation factors that should be taken into account if the medical health check for hand-intensive work is introduced in Swedish legislation. The process model will, in our studies, be used on hand-intensive work exposures due to the new regulation, in Sweden. Our belief is that the model, in the future, could also be used as a process model for other medical health checks, targeting other exposures in the work environment. The information from the different stakeholders involved in occupational health surveillance, in this case including the OHS-ergonomists, managers, and workers, are important sources of knowledge to be used in the further development of the specific HIW-model, as well as to identify what kind of support is needed for occupational health surveillance to be integrated into systematic work environment management. Furthermore, the stakeholders’ perceptions of the model in the different companies will, hopefully, give indications regarding how occupational health surveillance targeting hand-intensive work can be organized, together with occupational health surveillance targeting exposure to hand-arm vibrations.

An important strength of this study is the descriptive and explorative design, in which the process and its outcome shall reflect a service provided to each company by their OHS-provider, regarding both debit and execution of the service. The study population will consist of different companies, which will have a variety of exposure variables relevant for hand-intensive work, for example, force, repetition, and posture. Having this variety increases the transferability of the study [31], since it will indicate whether the medical health check for hand-intensive work is relevant for the different work sectors. 

A limitation of this study is the probability of selection bias. The participating companies will likely have a special interest in work environmental issues and the health of their employees. This limitation will be handled by interviewing several informants from the companies as well as the ergonomists, which together will give their individual experiences of the process. Moreover, in the evaluation of outcomes, also data from the workers’ questionnaires will be analyzed. Their perceptions are probably not colored by the company’s approach to work environment issues. However, a limitation is the relatively small sample size of workers in study 2, which will not yield enough statistical power to allow for the use of parametric inferential statistical methods. However, both descriptive statistics and non-parametric tests will be applied to investigate potential differences in perceived workload, and the prevalence of perceived pain between baseline and follow-ups. A possible alternative method would have been to also interview the workers, however for logistical reasons this will not be feasible. 

The response rate will decrease between each questionnaire collection point because of common events such as workers changing or quitting their work [40]. The period for follow-up is relatively short; thus, hopefully, the response losses will not be significantly affected. On the other hand, the short follow-up time for the questionnaires can be seen as a limitation, as changes in the work environment might take time to implement, and effects regarding pain variables might not have occurred yet. However, if work environmental actions are taken, the workers should be aware of those actions and give answers about what changes have been implemented. Those eventual changes ought to affect their perceptions of the exposure levels at their workplace. The relatively short follow-up period for the workers’ questionnaires is balanced by the relatively long period that will follow, between the interviews of company representatives in study 1, which explores the informant’s experiences of the HIW-model, and the interview in study 2, which will explore the outcomes regarding changes in the work environment, followed by the feedback from ergonomists after exposure assessments and medical health checks. 

## 5. Conclusions

The project is expected to generate knowledge regarding the value of the process model for occupational health surveillance. The project is anticipated to shed light on what factors facilitate or impede execution of the model, from the different stakeholders’ perspectives; the employer as having the legal responsibility for the work environment, and the occupational health service consultants, being the work environment experts supporting the employers. By utilizing the KTA-framework as guidance in the design of the research project, the end-users’ experiences are explored in order to gain knowledge of their needs, for the further development the HIW-model and supplementary guidelines.

Furthermore, the project is expected to contribute to existing knowledge regarding occupational health surveillance, regardless of the type of exposure, and the interconnection between exposure assessment and medical health checks.
